# The association between *ADAM12* gene polymorphisms and osteoarthritis: an updated meta-analysis

**DOI:** 10.1186/s13018-023-03626-7

**Published:** 2023-03-01

**Authors:** Su Yang, Yue-peng Wang, Xi-yong Li, Peng-yong Han, Peng-fei Han

**Affiliations:** 1 Department of Orthopedics, Heping Hospital Affiliated to Changzhi Medical College, Changzhi, Shanxi China; 2 Department of Graduate School, Changzhi Medical College, Changzhi, Shanxi China

**Keywords:** Osteoarthritis, ADAM12, Gene polymorphism, Meta-analysis, Systematic review

## Abstract

**Background:**

Osteoarthritis of the knee is an irreversible disease that causes great pain, and genetic factors play an important role in its occurrence and development. There have been many studies on the correlation between *ADAM12* polymorphisms and genetic susceptibility to osteoarthritis, but the results remain inconclusive.

**Methods:**

Papers from PubMed, Web of Science, EMbase, Springer, SCOPUS, Google Scholar and other databases were systematically retrieved with a cut-off of January 2022. All case–control studies on *ADAM12* rs3740199, rs1871054, rs1044122, and rs1278279 polymorphisms and osteoarthritis were searched. Fixed or random effects models were used for pooled analysis with OR values and 95% confidence intervals (CI), and publication bias was assessed. In addition, the false-positive reporting probability test was used to assess the confidence of a statistically significant association.

**Results:**

Eleven articles were included, which included 3332 patients with osteoarthritis and 5108 healthy controls. Meta-analysis showed that the rs1871054 polymorphism of *ADAM12* was associated with osteoarthritis in dominant, recessive, allelic, and homozygote genetic models [C vs. T: OR = 1.34 95% CI (1.05, 1.71), *P* < 0.001]. Our subgroup analysis revealed an association between the *ADAM12* polymorphism rs1871054 in Asians and osteoarthritis [C vs. T: OR = 1.61, 95% CI (1.25, 2.08), *P* < 0.001], albeit this was only for three studies. In addition, the *ADAM12* polymorphism rs1871054 is associated with osteoarthritis in patients younger than 60 years of age [C vs. T: OR = 1.39, 95% CI (1.01, 1.92), *P* = 0.289]; however, the *ADAM12* gene rs3740199, rs1044122, and rs1278279 site polymorphisms were not significantly. Furthermore, when assessing the confidence of the positive results, the positive results were found to be credible (except for Age < 60).

**Conclusion:**

Polymorphism at the rs1871054 site of *ADAM12* is associated with genetic susceptibility to osteoarthritis, but rs3740199, rs1044122, and rs1278279 site polymorphisms are not.

## Introduction

Osteoarthritis (OA) is a chronic progressive joint disease with increasing incidence with age. It can occur in any joint of the body and is characterized by damage to articular cartilage, subchondral sclerosis, and osteophyte formation [1]. Studies have shown that the global prevalence of OA of the knee or hip are 3.8% and 0.85%, respectively [2]. The prevalence of OA is expected to continue to increase in the future due to the aging of the global population, but the etiology of OA is still unknown. Many studies argue that OA is a multifactorial disease, and genetic and environmental factors (age, sex, obesity, physical activity, major trauma, occupation, etc.) are closely related to its development [3, 4]. In recent years, many researchers have attempted to explore the causes of OA at the genetic level, and a variety of genes that may be associated with OA have been identified, including *ADAM12*, vitamin D receptor, matrix metalloproteinases, estrogen receptor, and interleukins [5–8]. Currently, it has been found that the mutation of *IL-1Ra Ser133Ser* does not appear to be associated with immune-mediated and inflammatory diseases in a variety of genetic patterns, suggesting that the mutant allele C of *IL-1Ra Ser133Ser* does not increase the risk of disease [9]. Studies have also shown that mutations in inflammatory cytokines (G allele at rs361525, T allele at rs419598, and A allele at rs2228145) can lead to increased prevalence of coronavirus disease 2019 [10]. This provides more reliable and sound evidence for basic research and clinical treatment. In addition, since it is difficult to make early diagnosis of basic biomarkers with traditional biomarkers (tumor necrosis factor α, C-reactive protein, cytokines, etc.) [11], the research of new therapeutic options (nanotechnology [11, 12], targeted drugs [11], autophagy regulatory drugs [13], etc.) is also plagued by non-specific targets. Such studies could help in the search for new treatments.

*ADAM12* (a disintegrin and metalloproteinase domain 12) is an active protease that is mainly responsible for protein decomposition, adhesion, and signal transduction [14]. It belongs to the *ADAM* family and is involved in the formation and proliferation of chondrocytes and the differentiation of osteoclasts [15, 16]. Some studies have shown that *ADAM12* expression increases continuously in patients with OA [17, 18], and neutralizing antibodies against *ADAM12* can significantly reduce the degradation of oligomeric cartilage proteins [19]. Other studies have shown that *ADAM12* can promote the proliferation and maturation of chondrocytes by inhibiting insulin-like growth factor 1 (IGF-1) signaling pathway and regulating metalloproteinases and adhesion activities [16, 20]. When *ADAM12* genes is mutated, *ADAM12* is overexpressed in joints [21], and this protein overexpression induces inflammation by participating in cytokine receptor interactions and the osteoclast differentiation pathway [22]. In addition, *ADAM12* polymorphism affects the balance between synthesis and degradation of extracellular matrix (ECM), leading to degradation of chondrocytes [16, 20]. These evidences suggest that *ADAM12* is vital in the development of OA.

*ADAM12* is expressed in both normal and arthritic articular cartilage. The occurrence and development of OA can be affected by polymorphisms in the *ADAM12* gene, and the differences in protein activity and conformation caused by *ADAM12* may have varying effects on articular cartilage [5, 21, 23]. In recent years, *ADAM12* gene polymorphisms have become a hot topic in the search for genetic factors related to OA risk [24]. *ADAM12* mutation is also associated with the severity of OA [25]. So far, there is no consensus on whether *ADAM12* polymorphisms are related to disease susceptibility, severity, or phenotype. A study on Estonian patients suggested that *ADAM12* gene polymorphisms were closely associated with the occurrence of knee arthritis [26], and the rs1871054 intron C allele was considered to confer susceptibility to advanced OA. A recent Asian study showed similar results [24] and found that this was more likely to occur in female patients. A meta-analysis of *ADAM12* suggested a positive correlation with male sex of patients [27]. However, a European study found no significant association between *ADAM12* polymorphisms and knee OA [5]. There is still great controversy regarding the relationship between *ADAM12* polymorphisms and OA. Is the *ADAM12* gene related to OA and gender? In this study, a meta-analysis was conducted on the correlation between G/C, T/C, G/A, and T/C polymorphisms of rs3740199, rs1871054, rs1044122, and rs1278279 of *ADAM12* and OA, to evaluate whether *ADAM12* mutations are related to susceptibility to OA.

## Data and methods

### Literature retrieval strategy

We conducted a systematic search for case–control studies of *ADAM12* polymorphisms and OA on the Web of Science, PubMed, EMbase, Cochrane Library, SCOPUS, Google Scholar and other databases, with a cut-off of January 2022. There was no language restriction. We searched for keywords including “osteoarthritis,” “OA,” “degenerative joint disease,” “ADAM12,” “gene polymorphism," and “polymorphism”. All obtained studies were screened, and references to relevant articles were retrieved to make the included studies more accurate and comprehensive.

### Inclusion and exclusion criteria

Inclusion criteria: (1) Subjects were diagnosed with OA; (2) The study concerned the relationship between *ADAM12* polymorphisms and OA; (3) Allele or genotype distribution frequency data were available; (4) Genotypic distribution conformed to Hardy–Weinberg equilibrium (HWE). The exclusion criteria were as follows: (1) Repeated studies and publications; (2) Reviews of literature, case reports, and conference abstracts; (3) animal experiments; and (4) studies with a Newcastle–Ottawa scale (NOS) score < 6 [28].

### Literature screening

Data were independently extracted from all eligible studies by two researchers, cross-checked, and discussed with the assistance of a third researcher in cases of disagreement. Information was collected, including author, year of publication, study area, OA site, diagnostic criteria, genotyping method, total number of cases and controls, and *ADAM12* genotype frequency.

### Quality evaluation

Two researchers independently evaluated eligible studies using the Newcastle–Ottawa Scale (NOS) and discussed them in cases of disagreement. The NOS consists of three parts: study population selection (four items), intergroup comparability (one item), and measurement of exposure factors (three items). The full score was 9, and a score ≥ 6 was considered a high-quality study suitable for meta-analysis [28].

### Statistical analysis

All statistical analyses were performed using Stata 15.0 software, and the following five gene models were compared: (1) dominant (CC + GC vs. GG; CC + TC vs. TT; AA + GA vs. GG), (2) recessive (CC vs. GC + GG; CC vs. TC + TT; AA vs. GA + GG), (3) allelic (C vs. G; C vs. T; A vs. G), (4) homozygous (CC vs. GG; CC vs. TT; AA vs. GG), and (5) heterozygous (GC vs. GG; TC vs. TT; GA vs. GG). The correlations between *ADAM12* polymorphisms at the rs3740199, rs1871054, rs1044122, and rs1278279 sites (C/G, C/T, A/G, and C/T) and OA were evaluated by summarizing the OR value and 95% CI. The χ^2^ test was used to confirm whether the genotype frequencies of the included studies were consistent with HWE. Simultaneously, a subgroup analysis based on various regions was conducted to observe the influence of different regions of the results. Moreover, subgroup analysis was conducted to observe the effect of sex on the results. The Q test was used to determine statistical heterogeneity among studies [29]. When there was significant heterogeneity between studies (I^2^ > 50%), a random-effects model was used for data calculation [30]; when I^2^ < 50%, a fixed-effects model was used [31]. I^2^ < 25% indicated low heterogeneity; I^2^ value between 25 and 75% indicates moderate heterogeneity; I^2^ > 75% indicated high heterogeneity; if significant heterogeneity was found, sensitivity analysis was performed to explore its possible sources. Egger’s and Begg’s tests were used to detect publication bias. *P* > 0.05, indicating no obvious publication bias.

### False-positive report probability (FPRP) analysis

In this study, positive results from meta-analysis were further applied to FPRPS, which could help us explore the probability of a meaningful association between SNPS and disease [32, 33]. The FPRP threshold was set at 0.2, the prior probabilities were set at 0.25, 0.1, 0.01, 0.001 and 0.0001, and the correlation strength index OR = 1.5.

## Results

### Literature retrieval results

**Fig. 1:**
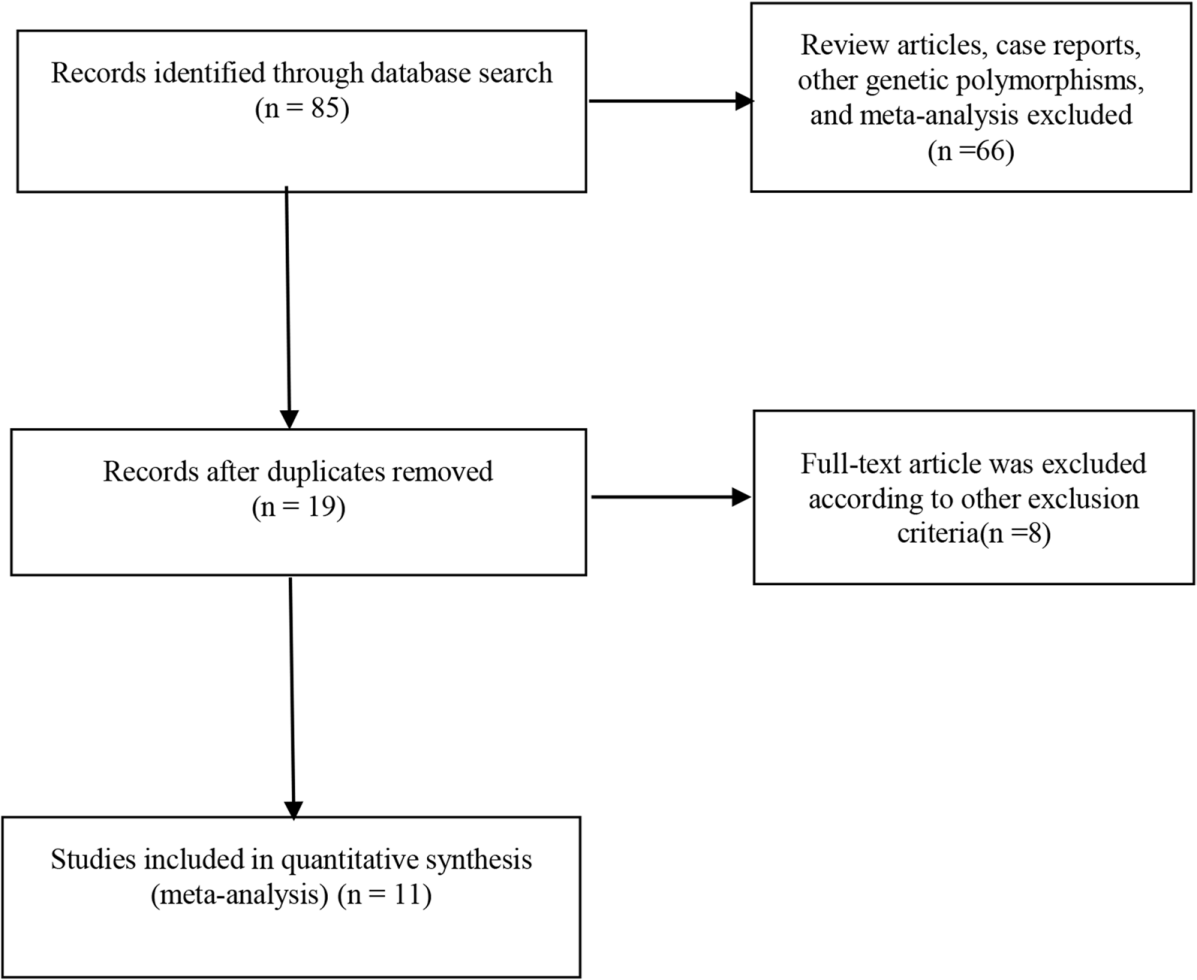
Flow diagram of the literature search

The databases were searched using a retrieval strategy. By reading the title and abstract, studies that may be consistent were preliminarily screened. We retrieved 27 studies were retrieved from the Web of Science database, 20 from PubMed, 20 from Springer, and 18 from Embase. Endnote software was used to remove 66 duplicate studies, and 9 reviews, non-case control studies, studies lacking genotypes [34–36], and those with incomplete data were removed from the remaining 19 studies, and the remaining 11 studies were suitable for the meta-analysis [5, 24–26, 37–42]. In the Literature screening, there was no disagreement among independent researchers about the included studies. A flowchart is shown in Fig. 1. A total of 8440 patients were included in the 11 studies, including 3332 patients with OA in the case group and 5108 patients without OA in the control group. The included studies were consistent with the H–W inheritance law.

Among the included studies, eight articles investigated the association between the rs3740199 polymorphism and OA in 1686 and 2845 patients in the case and control groups, respectively. Seven articles investigated the association between the rs1871054 polymorphism and OA in 1204 and 1425 patients in the case and control groups, respectively. The association between the rs1278279 polymorphism and OA was studied in 316 and 379 patients in the case and control groups from two articles. Finally, four studies investigated the relationship between the rs1044122 polymorphism and OA in 904 and 988 patients in the case and control groups, respectively. Four studies were conducted on European population [5, 26, 37, 41], among which Rodriguez-Lopez et al. [40] included three centers in Spain, Britain, and France, so it was considered an independent study. Five studies were conducted on Asian population [24, 25, 38, 39, 41]. Kerna et al. [26, 37] included tibial and patellar OA in their 2009 and 2013 studies; therefore, they were analyzed as two studies. One study focused on a North American population [42]; therefore, a subgroup analysis was not conducted. In addition, gender analysis was performed in four studies [24, 26, 37, 38] and not in the rest. The basic characteristics and distribution of the alleles and genotypes of the included studies are shown in Tables 1 and 2. All included studies were of high quality, with NOS scores ≥ 6, as shown in Table 1.

**Table 1:** Characteristics of studies included in the meta-analysis of overall OA

Study author	Year	Country	Ethnicity	Sex	Number of samples			Age case/control (mean)	Genotyping method	Quality score
					Cases	Controls	Total			
Kerna et al. (1) [37]	2009	Estonian	European	Male	40	60	100	32–55/NR	PCR–RFLP	8
				Female	123	155	278	32–55/NR		
Rodriguez-Lopez et al. [40]	2009	Spain	European	Male	51	179	230	68 (55–84)/68 (55–94)	Multiplex PCR	6
				Female	211	115	326	68 (55–84)/68 (55–94)		
		UK	European	Male	164	342	506	65 (55–85)/69 (55–89)	Multiplex PCR	6
				Female	196	356	552	65 (55–85)/69 (55–89)		
		Greece	European	Male	20	56	76	72 (62–85)/70 (46–88)	Multiplex PCR	6
				Female	139	137	276	72 (62–85)/70 (46–88)		
Valdes et al	2004	UK	European	Female	280	469	749	43–67/NR	PCR–SSCP	5
Shin et al. [38]	2012	Korean	Asian	Male	171	882	1053	67.4 ± 7.9/62.7 ± 7.9	TaqMan	7
				Female	554	855	1409	67.4 ± 7.9/62.7 ± 7.9		
Kerna et al. (2) [26]	2013	Estonian	European	Male	54	66	120	44.35.9/46.1 (5.5)	TaqMan	8
				Female	131	143	274	44.35.9/46.1 (5.5)		
Lou et al. [39]	2014	China	Asian	Male	58	77	135	63.1 ± 5.2/62.2 ± 4.2	TaqMan	9
				Female	94	102	196	63.1 ± 5.2/62.2 ± 4.2		
Wang et al. [25]	2015	China	Asian	Male	58	62	120	67.4 ± 4.2/65.9 ± 5.3	iMLDR	9
				Female	106	138	244	67.4 ± 4.2/65.9 ± 5.3		
Poonpet et al. [41]	2016	Thai	Asian	Male	53	51	104	69.0 ± 8.2/57.3 ± 5.8	HRM-SNP	7
				Female	147	149	296	69.0 ± 8.2/57.3 ± 5.8		
Aguilar et al. [42]	2020	Mexico	North America	Male	46	68	114	63.53 ± 14.52/55.98 ± 13.13	TaqMan	8
				Female	86	96	182	63.53 ± 14.52/55.98 ± 13.13		
Haberal et al. [5]	2021	Turkish	European	Male	42	26	48	66 ± 9.8/57.3 ± 11.2	Real-time PCR	8
				Female	108	124	232	66 ± 9.8/57.3 ± 11.2		
Fatima et al. [24]	2022	Pakistan	Asian	Male	88	100	188	50.45 ± 0.49/49.13 ± 0.53	ARMS‐PCR	8
				Female	312	300	612	50.45 ± 0.49/49.13 ± 0.53		

*NR* Not reported

**Table 2:** Distribution of genotype and allele among OA patients and controls

Study author	Sex	Patient group					Control group					P-HWE	MAF
		GG	GC	CC	G	C	GG	GC	CC	G	C		
*Rs3740199*													
Kerna [37]^a^	Male	2	7	6	11	19	4	15	16	23	47	0.87	0.67
	Female	4	25	22	33	69	8	31	49	47	129	0.35	0.73
Kerna [37]^b^	Male	1	8	16	10	40	5	13	7	23	27	0.82	0.54
	Female	9	26	37	34	100	3	30	34	36	98	0.25	0.73
Shin [38]	Male	45	94	32	184	158	281	423	178	985	779	0.41	0.44
	Female	169	270	115	608	500	243	440	172	926	784	0.29	0.46
Poonpet [41]	Male/Female	42	102	56	186	214	54	100	46	208	192	0.98	0.48
Wang [25]	Male/Female	44	84	36	172	156	51	102	47	204	196	0.77	0.50
Lou [39]	Male/Female	42	78	32	162	142	44	93	42	181	177	0.60	0.50
Haberal [5]	Male/Female	51	76	23	178	122	38	78	34	154	146	0.62	0.49
Aguilar [42]	Male/Female	58	45	29	161	103	67	76	21	210	118	0.94	0.36
Rodriguez-Lopez [40]*	Male/Female	NA	NA	NA	234	290	NA	NA	NA	261	327	NA	0.44
Rodriguez-Lopez [40]*	Male/Female	NA	NA	NA	350	370	NA	NA	NA	652	744	NA	0.47
Rodriguez-Lopez [40]*	Male/Female	NA	NA	NA	138	180	NA	NA	NA	147	239	NA	0.38

Study author	Sex	Patient group					Control group					P-HWE	MAF
		TT	TC	CC	T	C	TT	TC	CC	T	C		
*Rs1871054*													
Lou [39]	Male/Female	26	57	69	109	195	47	88	44	182	176	0.83	0.50
Wang [25]	Male/Female	29	59	76	117	211	52	99	49	203	197	0.89	0.49
Haberal [5]	Male/Female	43	67	40	153	147	41	70	39	152	148	0.41	0.49
Aguilar [42]	Male/Female	24	76	32	124	140	21	90	53	132	196	0.07	0.60
Kerna [37]^a^	Male	4	8	3	16	14	10	17	8	37	33	0.88	0.47
	Female	14	25	12	53	49	20	45	23	85	91	0.82	0.52
Kerna [37]^b^	Male	7	11	7	25	25	7	14	4	28	22	0.50	0.44
	Female	17	35	20	69	75	17	35	15	69	65	0.71	0.49
Kerna [26]	Male	3	7	10	13	27	14	29	8	57	45	0.27	0.45
Kerna [26]^a^	Male	2	3	8	7	19	19	35	12	73	59	0.55	0.55
Fatima [24]	Male	35	37	26	107	89	35	41	24	447	353	0.09	0.45
	Female	71	153	88	295	329	97	142	61	336	264	0.49	0.44

Study author	Sex	Patient group					Control group					P-HWE	MAF
		GG	GA	AA	G	A	GG	GA	AA	G	A		
*Rs1278279*													
Wang [25]	Male/Female	92	62	10	264	82	121	64	15	306	94	0.12	0.24
Lou [39]	Male/Female	84	59	9	238	77	106	60	13	272	86	0.27	0.24

Study author	Sex	Patient group					Control group					P-HWE	MAF
		TT	TC	CC	T	C	TT	TC	CC	T	C		
*Rs1044122*													
Wang [25]	Male/Female	51	88	25	190	138	62	101	37	225	175	0.71	0.44
Lou [39]	Male/Female	47	81	24	129	175	56	92	31	204	154	0.52	0.43
Kerna [26]	Male	21	27	6	69	39	29	27	10	85	47	0.38	0.37
	Female	58	65	8	181	81	53	66	24	172	114	0.66	0.40
Fatima [24]	Male	40	39	9	119	57	53	39	8	145	55	0.83	0.28
	Female	125	144	46	394	236	172	102	26	446	154	0.06	0.27

*P-HWE* *P*-value for Hardy–Weinberg equilibrium; *MAF* Minor allele frequency of control group; *NA* Data not available

^a^: tibiofemoral knee OA; ^b^: patellofemoral knee OA; *: An independent study in one article

### Meta-analysis results

#### Heterogeneity and publication bias

Heterogeneity was analyzed for all genotypes, and it was found that there was no significant heterogeneity in any of the rs3740199 and rs1278279 polymorphisms of *ADAM12*. A fixed-effects model was used for data calculation. However, there was significant heterogeneity in the polymorphism studies of rs1871054 (CC + TC vs. TT, C vs. T, CC vs. TT) and rs1044122 (all gene models), so a random-effects model was used for data calculation. At the same time, sensitivity analysis was conducted to observe the impact of each study on the overall results by ignoring one study at a time and verifying the stability of the pooled results. The results showed that the I^2^ value changed from > 50 to < 50% when the research results of Fatima et al. [24] were ignored in rs1044122, so the source of heterogeneity was the research of Fatima et al. [24]. Sensitivity analysis of rs1871054 for *ADAM12* did not identify the source of heterogeneity. Through careful reading by Fatima et al. [24], it was found that the inclusion of the case group and the control group met the inclusion criteria, and the *ADAM12* gene was also determined by arms-PCR technology. No obvious abnormality was found in the experimental method, and the distribution of genotype data met Hardy Weinberg equilibrium law. However, this study is the only one on Pakistanis. Mukhtar et al. [43] mentioned that 70% of marriages in Pakistan are sincere marriages, which is the main reason for the inheritance of genetic diseases to the next generation, so this may be the source of the existence of heterogeneity. Egger’s and Begg’s tests were carried out for all genotypes, and no obvious publication bias was found, indicating that the results were relatively stable. The *P* values of the publication bias test for the included polymorphisms of each genotype are shown in Table 3.

**Table 3:** The results were summarized in the meta-analysis of *ADAM12* gene polymorphisms in association with knee osteoarthritis risk

Subgroup	Genetic model	Sample size	Test of association		Test of heterogeneity		Test of publication bias (Begg’s test)		Test of publication bias (Egger’s test)	
		Case/control	OR	95% CI (*P*-value)	*I*^2^ (%)	*P*	*Z*	*P*	*T*	*P*
*Rs3740199*										
Overall	Dominant model	1686/2845	0.98	0.86–1.13 (0.798)	0	0.585	0.87	0.386	− 0.75	0.479
	Recessive model	1686/2845	1.02	0.88–1.18 (0.778)	43.0	0.092	0.12	0.902	0.05	0.960
	Allelic model	2467/4030	0.98	0.91–1.05 (0.567)	28.4	0.174	0.16	0.876	− 0.21	0.841
	CC versus GG	1686/2845	1.01	0.85–1.21 (0.884)	22.8	0.248	0.87	0.386	− 0.32	0.761
	GC versus GG	1686/2845	0.97	0.84–1.12 (0.664)	0	0.535	0.87	0.386	− 0.86	0.424
Subgroup										
European	Dominant model	313/365	0.74	0.49–1.11 (0.149)	0	0.677	1.04	0.296	2.68	0.232
	Recessive model	313/365	0.86	0.61–1.20 (0.374)	64.2	0.061	0.00	1.000	− 1.27	0.424
	Allelic model	1094/1550	0.91	0.82–1.02 (0.111)	37.8	0.154	0.00	1.000	0.12	0.908
	CC versus GG	313/365	0.67	0.41–1.10 (0.115)	0	0.451	0.00	1.000	3.19	0.193
	GC versus GG	313/365	0.79	0.51–1.21 (0.275)	0	0.513	1.04	0.296	0.56	0.673
Asian	Dominant model	1241/2316	1.04	0.85–1.25 (0.656)	0	0.502	1.02	0.308	0.01	0.995
	Recessive model	1241/2316	1.02	0.86–1.21 (0.843)	0	0.642	1.02	0.308	− 0.03	0.978
	Allelic model	1241/2316	1.02	0.92–1.13 (0.689)	0	0.398	1.02	0.308	− 0.01	0.991
	CC versus GG	1241/2316	1.04	0.85–1.27 (0.703)	0.4	0.390	1.02	0.308	− 0.01	0.995
	GC versus GG	1241/2316	1.04	0.88–1.22 (0.681)	0	0.709	1.02	0.308	− 0.01	0.990
Male	Dominant model	211/942	1.16	0.75–1.80 (0.494)	0	0.766	0.00	1.000	0.49	0.709
	Recessive model	211/942	1.07	0.74–1.53 (0.735)	69.1	0.039	0.00	1.000	0.74	0.593
	Allelic model	211/942	1.15	0.93–1.43 (0.201)	68.9	0.040	0.00	1.000	0.60	0.656
	CC versus GG	211/942	1.25	0.79–1.97 (0.340)	49.2	0.140	1.04	0.296	0.67	0.623
	GC versus GG	211/942	1.11	0.78–1.58 (0.565)	65.2	0.057	0.00	1.000	0.27	0.832
Female	Dominant model	677/1010	1.38	1.02–1.86 (0.037)	36.1	0.209	1.04	0.296	− 0.56	0.675
	Recessive model	677/1010	0.98	0.77–1.23 (0.843)	3.6	0.354	1.04	0.296	− 0.96	0.513
	Allelic model	677/1010	0.96	0.84–1.11 (0.590)	0	0.629	0.00	1.000	− 0.36	0.777
	CC versus GG	677/1010	0.91	0.68–1.22 (0.534)	0	0.404	1.04	0.296	− 1.13	0.461
	GC versus GG	677/1010	1.00	0.83–1.20 (0.969)	0	0.869	0.00	1.000	− 0.27	0.833
Age < 60	Dominant model	163/215	0.94	0.47–1.90 (0.864)	0	0.713	0.00	1.000	No	No
	Recessive model	163/215	1.00	0.67–1.51 (0.988)	64.2	0.039	0.00	1.000	No	No
	Allelic model	163/215	1.09	0.79–1.51 (0.587)	73.0	0.054	0.00	1.000	No	No
	CC versus GG	163/215	0.95	0.45–1.99 (0.891)	0	0.809	0.00	1.000	No	No
	GC versus GG	163/215	0.93	0.45–1.93 (0.840)	6.6	0.301	0.00	1.000	No	No
Age ≥ 60	Dominant model	1523/2630	0.98	0.86–1.13 (0.821)	8.6	0.362	1.88	0.060	− 0.79	0.475
	Recessive model	1523/2630	1.02	0.87–1.20 (0.813)	31.9	0.184	0.38	0.707	0.12	0.909
	Allelic model	2304/3815	0.97	0.90–1.05 (0.476)	18.4	0.279	0.73	0.466	− 0.69	0.510
	CC versus GG	1523/2630	1.02	0.85–1.22 (0.853)	44.3	0.110	1.13	0.260	− 0.23	0.828
	GC versus GG	1523/2630	0.97	0.83–1.13 (0.688)	0	0.421	1.50	0.133	− 1.03	0.360
*Rs1871054*										
Overall	Dominant model	1204/1425	**1.26**	**1.05–1.51 (0.013)**	24.8	0.223	0.10	0.917	− 0.17	0.869
	Recessive model	1204/1425	**1.68**	**1.13–2.51 (0.011)**	77.4	< 0.001	− 0.10	1.000	0.79	0.458
	Allelic model	1204/1425	**1.34**	**1.05–1.71 (0.019)**	76.4	< 0.001	0.10	0.917	0.53	0.610
	CC versus TT	1204/1425	**1.62**	**1.05–2.50 (0.031)**	69.1	0.001	0.31	0.754	0.34	0.743
	TC versus TT	1204/1425	1.07	0.88–1.30 (0.506)	0	0.907	0.31	0.754	− 1.86	0.106
Subgroup										
European	Dominant model	346/428	1.05	0.76–1.45 (0.787)	0	0.660	0.73	0.462	3.94	0.029
	Recessive model	346/428	1.85	0.94–3.63 (0.075)	72.5	0.006	1.71	0.086	3.78	0.032
	Allelic model	346/428	1.33	0.91–1.95 (0.145)	66.0	0.019	1.22	0.221	4.29	0.023
	CC versus TT	346/428	1.56	0.81–3.01 (0.187)	55.4	0.062	0.73	0.462	3.68	0.035
	TC versus TT	346/428	0.92	0.65–1.31 (0.63)	0	0.999	0.24	0.806	0.43	0.697
Asian	Dominant model	726/779	**1.52**	**1.20–1.92 (0.001)**	0	0.773	1.04	0.296	8.36	0.076
	Recessive model	726/779	**2.08**	**1.35–3.20 (0.001)**	70.3	0.034	0.00	1.000	6.03	0.105
	Allelic model	726/779	**1.61**	**1.25–2.08 (< 0.001)**	65.6	0.055	0.00	1.000	13.38	0.047
	CC versus TT	726/779	**2.21**	**1.52–3.22 (< 0.001)**	38.1	0.199	0.00	1.000	13.63	0.047
	TC versus TT	726/779	1.22	0.95–1.57 (0.125)	0	0.836	0.00	1.000	− 2.07	0.287
Male	Dominant model	138/160	1.16	0.75–1.80 (0.494)	0	0.766	1.71	0.086	1.91	0.152
	Recessive model	138/160	2.32	1.01–5.35 (0.049)	63.1	0.029	− 0.24	1.000	1.00	0.390
	Allelic model	138/160	1.52	0.95–2.43 (0.080)	57.2	0.053	0.73	0.462	1.57	0.215
	CC versus TT	138/160	2.02	0.91–4.47 (0.085)	39.6	0.157	− 0.24	1.000	1.43	0.248
	TC versus TT	138/160	0.93	0.57–1.51 (0.767)	0	0.992	− 0.24	1.000	0.42	0.702
Female	Dominant model	435/455	1.38	1.02–1.86 (0.037)	36.1	0.209	1.04	0.296	− 2.94	0.210
	Recessive model	435/455	1.38	1.01–1.88 (0.042)	0	0.448	1.04	0.296	− 1.49	0.376
	Allelic model	435/455	1.20	0.90–1.60 (0.220)	42.7	0.174	1.04	0.296	− 2.22	0.270
	CC versus TT	435/455	1.43	0.82–2.50 (0.211)	60.8	0.003	1.04	0.296	− 2.10	0.283
	TC versus TT	435/455	1.27	0.92–1.75 (0.143)	5.0	0.349	1.04	0.296	− 4.05	0.154
Age < 60	Dominant model	211/942	**1.31**	**1.02–1.68 (0.033)**	0	0.562	0.24	0.806	0.09	0.930
	Recessive model	211/942	**1.91**	**1.07–3.40 (0.029)**	68.7	0.012	1.22	0.221	1.40	0.255
	Allelic model	211/942	**1.39**	**1.01–1.92 (0.286)**	61.4	0.035	0.73	0.462	0.85	0.459
	CC versus GG	211/942	**1.74**	**1.00–3.02 (0.049)**	48.5	0.100	0.24	0.806	0.85	0.470
	GC versus GG	211/942	1.16	0.89–1.51 (0.285)	0	0.835	0.24	0.806	− 1.78	0.173
Age ≥ 60	Dominant model	677/1010	1.20	0.92–1.57 (0.172)	59.9	0.058	− 0.34	1.000	− 1.03	0.413
	Recessive model	677/1010	1.49	0.77–2.89 (0.241)	86.7	< 0.001	1.70	0.089	− 4.09	0.055
	Allelic model	677/1010	1.27	0.82–1.97 (0.286)	87.3	< 0.001	1.70	0.089	− 2.65	0.118
	CC versus GG	677/1010	1.45	0.67–3.14 (0.349)	83.5	< 0.001	1.70	0.089	− 2.14	0.166
	GC versus GG	677/1010	0.97	0.73–1.30 (0.854)	0	0.749	− 0.34	1.000	− 1.02	0.415
*Rs1278279*										
Overall	Dominant model	316/379	1.19	0.88–1.61 (0.265)	0	0.950	0.00	1.000	No	No
	Recessive model	316/379	0.80	0.44–1.47 (0.474)	0	0.995	0.00	1.000	No	No
	Allelic model	316/379	1.02	0.80–1.30 (0.893)	0	0.962	0.00	1.000	No	No
	AA versus GG	316/379	0.88	0.47–1.62 (0.671)	0	0.995	0.00	1.000	No	No
	GA versus GG	316/379	1.26	0.91–1.73 (0.158)	0	0.935	0.00	1.000	No	No
*Rs1044122*										
Overall	Dominant model	904/988	1.15	0.77–1.72 (0.479)	76.0	0.006	− 0.34	1.000	− 3.17	0.087
	Recessive model	904/988	0.87	0.49–1.54 (0.634)	76.4	0.005	1.02	0.308	− 6.22	0.025
	Allelic model	904/988	1.20	0.81–1.79 (0.359)	88.1	< 0.001	0.00	1.000	− 0.77	0.520
	CC versus TT	904/988	0.96	0.48–1.93 (0.877)	81.9	0.001	1.02	0.308	− 4.92	0.039
	TC versus TT	904/988	1.24	0.91–1.68 (0.179)	55.8	0.079	− 0.34	1.000	− 4.64	0.044
Subgroup										
Male	Dominant model	142/166	1.31	0.83–2.05 (0.248)	0	0.843	0.00	1.000	No	No
	Recessive model	142/166	0.98	0.47–2.05 (0.962)	0	0.404	0.00	1.000	No	No
	Allelic model	142/166	1.16	0.82–1.63 (0.398)	0	0.549	0.00	1.000	No	No
	CC versus TT	142/166	1.15	0.53–2.48 (0.727)	0	0.459	0.00	1.000	No	No
	TC versus TT	142/166	1.35	0.84–2.17 (0.222)	0	0.934	0.00	1.000	No	No
Female	Dominant model	446/443	1.25	0.46–3.38 (0.658)	91.5	0.001	0.00	1.000	No	No
	Recessive model	446/443	0.79	0.15–4.26 (0.782)	91.6	0.001	0.00	1.000	No	No
	Allelic model	446/443	1.09	0.43–2.75 (0.852)	94.6	< 0.001	0.00	1.000	No	No
	CC versus TT	446/443	0.89	0.12–6.83 (0.909)	93.6	< 0.001	0.00	1.000	No	No
	TC versus TT	446/443	1.35	0.64–2.87 (0.430)	83.6	0.014	0.00	1.000	No	No
Age < 60	Dominant model	211/942	1.29	0.61–2.71 (0.509)	89.2	0.002	0.00	1.000	No	No
	Recessive model	211/942	0.88	0.22–3.40 (0.835)	91.5	0.001	0.00	1.000	No	No
	Allelic model	211/942	1.12	0.54–2.33 (0.760)	93.8	< 0.001	0.00	1.000	No	No
	CC versus GG	211/942	0.99	0.20–4.96 (0.991)	93.2	< 0.001	0.00	1.000	No	No
	GC versus GG	211/942	1.38	0.81–2.34 (0.238)	76.5	0.039	0.00	1.000	No	No
Age ≥ 60	Dominant model	677/1010	1.01	0.73–1.39 (0.972)	0	0.948	0.00	1.000	No	No
	Recessive model	677/1010	0.84	0.56–1.26 (0.394)	0	0.767	0.00	1.000	No	No
	Allelic model	677/1010	1.29	0.68–2.48 (0.432)	88.9	0.003	0.00	1.000	No	No
	CC versus GG	677/1010	0.87	0.56–1.37 (0.542)	0	0.803	0.00	1.000	No	No
	GC versus GG	677/1010	1.05	0.75–1.48 (0.759)	0	0.978	0.00	1.000	No	No

Statistical significance values are shown in bold

*NO* No date obtained

#### Correlation between *ADAM12* polymorphism rs3740199 and osteoarthritis

**Fig. 2:**
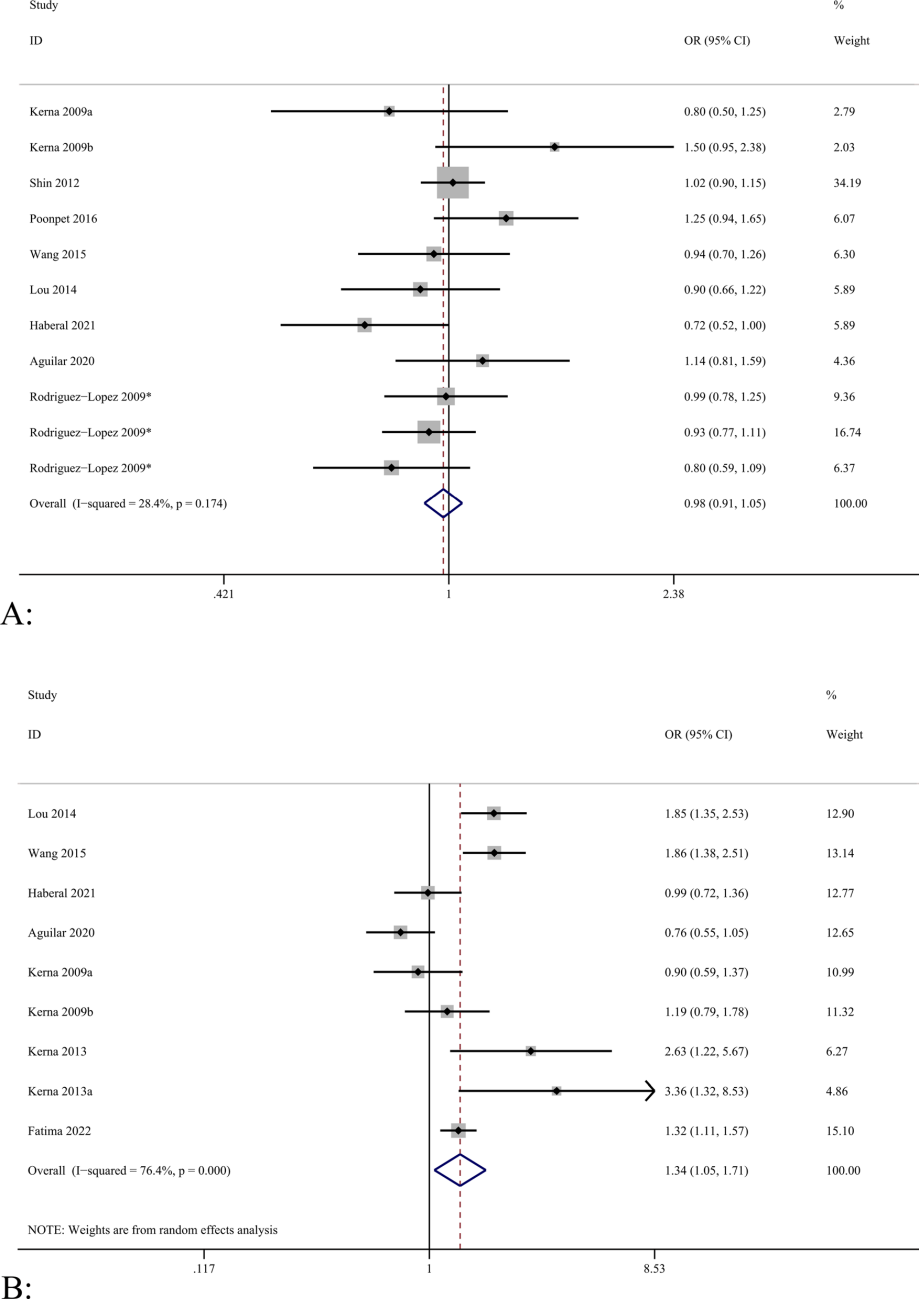

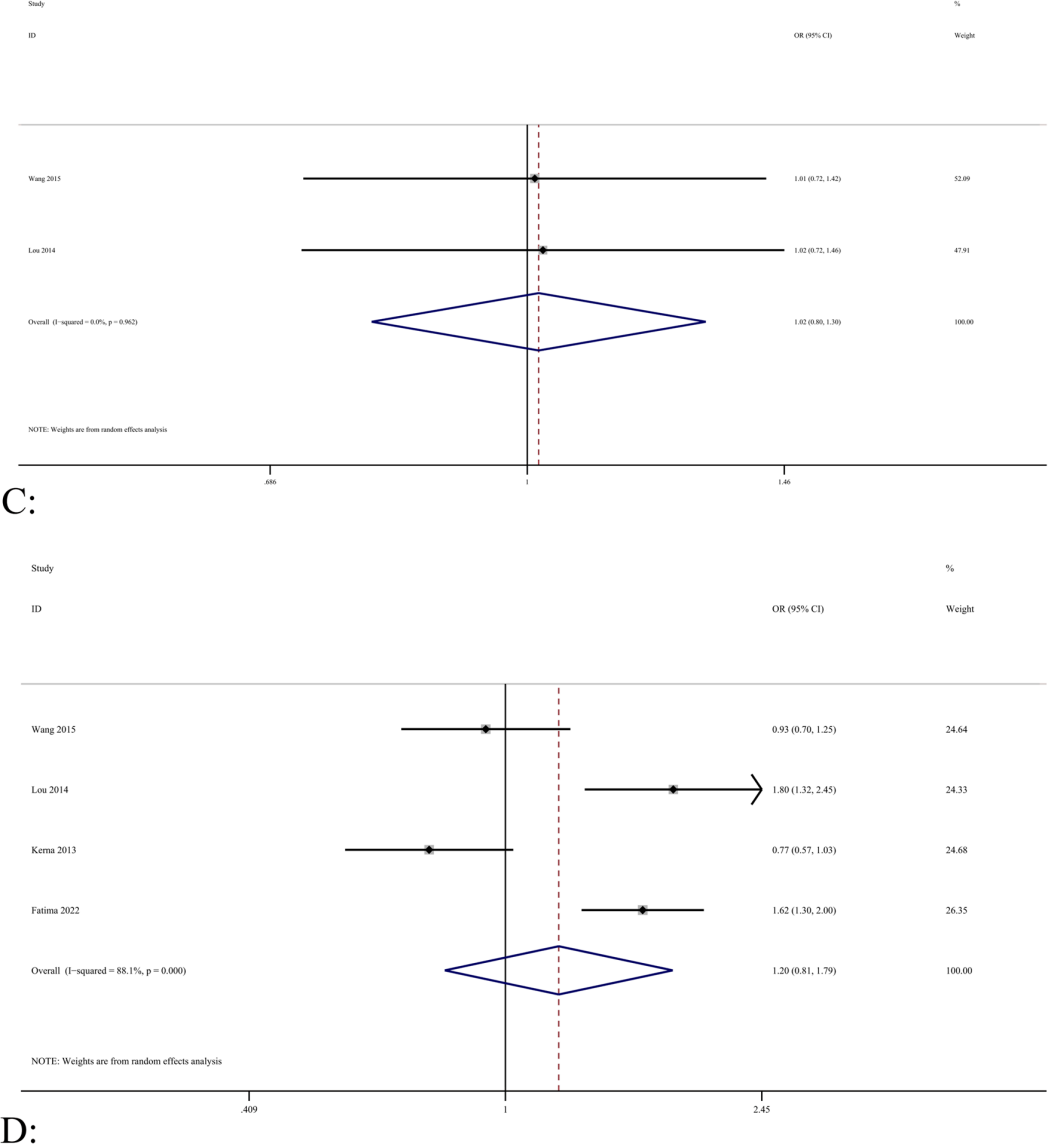
**A**–**D** show the forest maps of correlations between *ADAM12* rs3740199, rs1871054, rs1278279 and rs1044122 polymorphisms and osteoarthritis

A total of eight included studies focused on the correlation between the *ADAM12* rs3740199 polymorphism and OA [5, 25, 37–42], among which Rodriguez-Lopez et al. [40] included three centers in Spain, Britain, and France; therefore, the analysis was carried out across three cohorts, but the data in this study were incomplete. Kerna et al. [37] included tibial and patellar OA in their study; therefore, we treated them as two studies for our analysis. A total of 2467 and 4030 cases in the case and control groups were analyzed. The meta-analysis results showed that all gene models of the *ADAM12* rs1044122 polymorphism had no significant correlation with susceptibility to OA, and the comparative heterogeneity of all gene models was small [allelic model (C vs. G), OR = 0.98, 95% CI (0.91–1.05), *P* = 0.174, I^2^ = 28.4%], as shown in Fig. 2A. Publication bias was detected using the Begg’s test (*P* = 0.876) and Egger’s test (*P* = 0.841), and the results showed that the analysis results were fairly robust without obvious bias. No statistical significance was found in the other gene models (CC + GC vs. GG, CC vs. GC + GG, CC vs. GG, GC vs. GG).

According to regional subgroup analysis, there were four studies on Asians and six studies on Europeans, but only one study on North Americans, so subgroup analysis was not carried out on the North American population. The results showed that there were no significant differences in the alleles of *ADAM12* rs3740199 and OA susceptibility between Europeans and Asians. In addition, in the subgroup analysis based on sex, six studies were not statistically analyzed according to sex; therefore, only three studies were subjected to sex subgroup analysis, and the results were only meaningful in the female-dominant model [OR = 1.38, 95% CI (1.02–1.86), *P* = 0.209, I^2^ = 36.1%]. This does not seem to indicate that female patients are more susceptible to the disease. Subgroup analysis with an average age of 60 years indicated that the rs3740199 polymorphism of *ADAM12* and susceptibility to OA may not be significantly associated with age, as shown in Table 3.

#### Correlation between *ADAM12* polymorphism rs1871054 and osteoarthritis

**Fig. 3:**
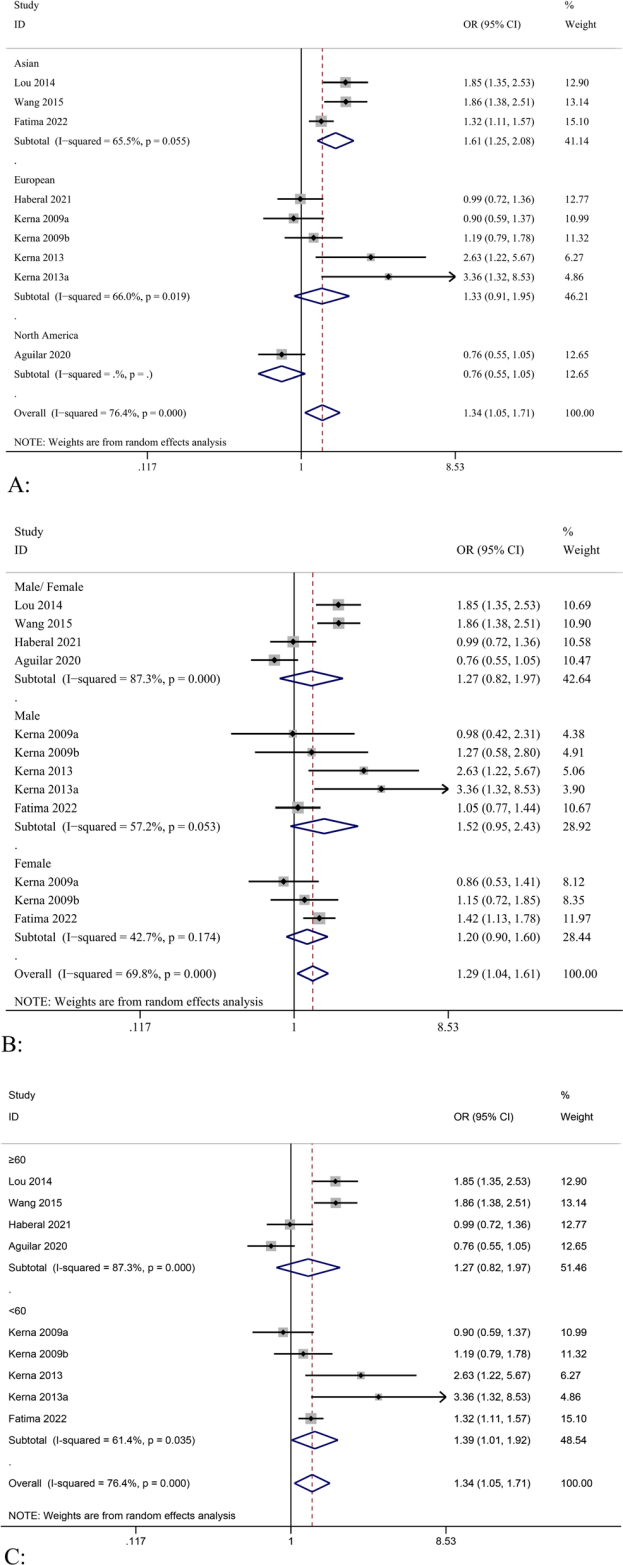

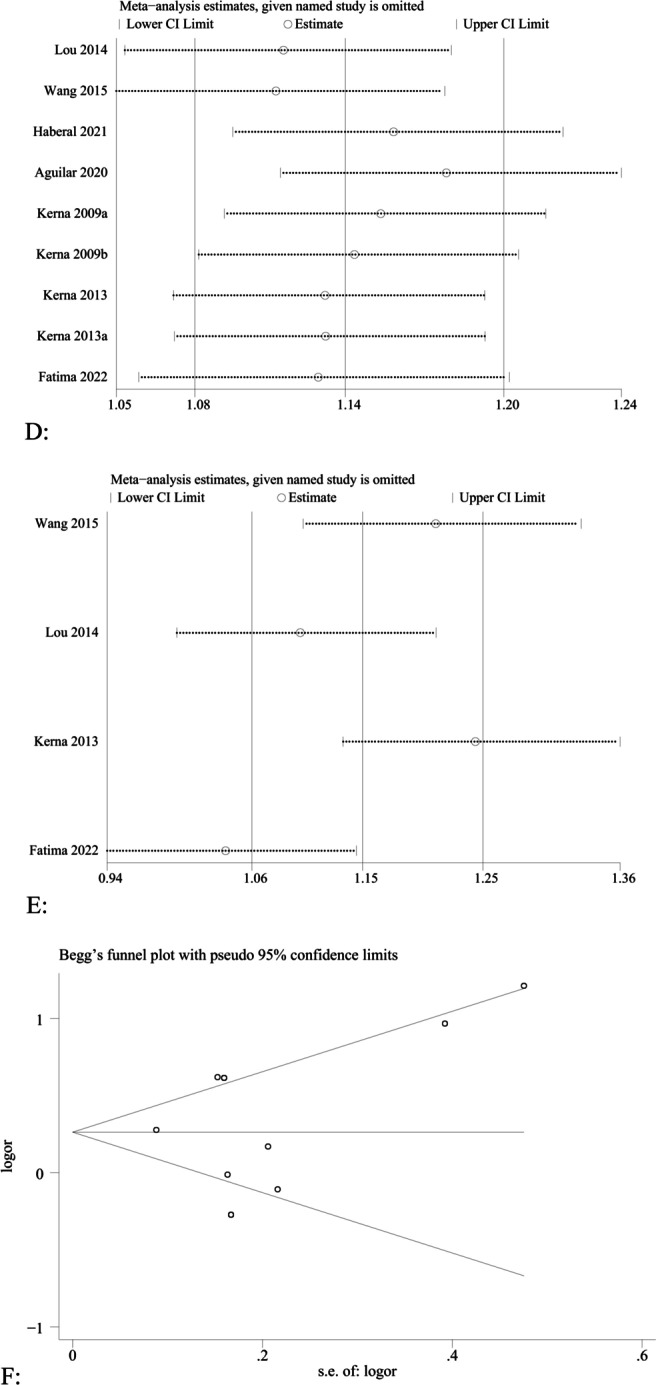
**A** Forest map analysis of different regional subgroups related to *ADAM12* rs1871054 (C vs. T) polymorphism and osteoarthritis. **B** Forest map analysis of different sex subgroups related to *ADAM12* rs1871054 (C vs. T) polymorphism and osteoarthritis. **C** Forest map analysis of different age subgroups related to *ADAM12* rs1871054 (C vs. T) polymorphism and osteoarthritis. **D** Sensitivity analysis of *ADAM12* rs1871054 (C vs. T) polymorphism and osteoarthritis. **E** Sensitivity analysis of *ADAM12* rs1044122 (C vs. T) polymorphism and osteoarthritis. **F** Begg’s test for publication bias

The association between the rs1871054 polymorphism of *ADAM12* and OA was analyzed in seven studies [5, 24–26, 37, 39, 42], which included 1204 and 1425 patients in the case and control groups, respectively. Kerna et al. [26, 37] included tibial and patellar OA in their study; therefore, the analysis was performed according to two studies. We found no statistically significant differences between *ADAM12* Rs1871054 polymorphism and susceptibility to osteoarthritis in the dominant, recessive, allele, and homozygous models [allelic model (T vs. C), OR = 1.34, 95% CI (1.05–1.71), *P* < 0.001, I^2^ = 76.4%], as shown in Fig. 2B. However, there was no statistical significance in the heterozygous model [OR = 1.07, 95% CI (0.88–1.30), *P* = 0.907, I^2^ = 0%], suggesting patients carrying the TC allele and those with TT had the same risk of disease. In summary, patients with the C allele may be more susceptible, which is consistent with the Kerna result [26]. Due to the large heterogeneity, no source of heterogeneity was found after the sensitivity analysis (Fig. 3D). In the allele model, Begg’s test (*P* = 0.917) (Fig. 3F), and Egger’s test (*P* = 0.610) were used to detect publication bias; the results showed that the analysis was fairly robust, without obvious bias.

Subgroup analysis showed that there was a significant difference between *ADAM12* rs1871054 and osteoarthritis susceptibility in Asian samples of dominant, recessive, allelic, and homozygous models [e.g., allelic model (T vs. C), OR = 1.61, 95% CI (1.25–2.08), *P* = 0.055, I^2^ = 65.6%] (Fig. 3A); however, only three studies were included. There was no significant difference between *ADAM12* rs1871054 in Europeans and the allele conferring susceptibility to osteoarthritis in all gene models [allelic model (T vs. C), OR = 1.33, 95% CI (0.91–1.95), *P* = 0.019, I^2^ = 66.0%], suggesting that Asian patients carrying the C allele are more prone to the disease. In addition, in the subgroup analysis based on sex, four studies were not statistically analyzed based on sex, so only three studies were analyzed based on sex subgroups. Kerna et al. [26] only provided data on male patients in their 2013 study. The results showed that *ADAM12* gene polymorphism was significant in male recessive models [OR = 1.38, 95% CI (1.02–1.86), *P* = 0.209, I^2^ = 36.1%] (Fig. 3B) and in female dominant and recessive models [e.g., dominant model (CC + GG vs. GG), OR = 1.38, 95% CI (1.02–1.86), *P* = 0.209, I^2^ = 36.1%]. However, it is uncertain whether *ADAM12* polymorphism is associated with OA susceptibility in either male or female patients. Subgroup analysis with a mean age of 60 years showed similar results to subgroup analysis by region, in which the C allele may be associated with disease susceptibility in patients younger than 60 years (Fig. 3C), as presented in Table 3.

#### Association between *ADAM12* polymorphism rs1278279 and osteoarthritis

Two studies on the correlation between the *ADAM12* polymorphism rs1278279 and OA were conducted on 316 and 379 patients in the case and control groups, respectively [25, 39], and the results showed that there was no significant difference between the rs1278279 alleles of *ADAM12* and OA susceptibility [e.g., allelic model (A vs. G), OR = 1.02, 95% CI (0.80–1.30), *P* = 0.926, I^2^ = 0%] (Fig. 3C). As there were only two studies, no subgroup analysis was performed, as shown in Table 3.

#### Correlation between *ADAM12* polymorphism rs1044122 and osteoarthritis

The correlation between the *ADAM12* rs1044122 polymorphism and OA was assessed in four studies, which included 904 and 988 patients in the case and control groups, respectively [24–26, 39]. The meta-analysis results showed that the rs1044122 polymorphism was not significantly correlated with susceptibility to OA, and the comparative heterogeneity of all gene models was large [e.g., allelic model (C vs. T), OR = 1.20, 95% CI (0.81–1.79), *P* < 0.001, I^2^ = 88.1%] (Fig. 2D). The sensitivity analysis showed that the source of heterogeneity was the study of Fatima et al. [24] (Fig. 3E). Begg’s (*P* = 1.000) and Egger’s tests (*P* = 0.520) were used to detect publication bias, and the results showed that the analysis was fairly robust without obvious bias. Since all four articles were conducted on Asian populations, subgroup analysis was not performed. In addition, in the subgroup analysis based on sex, only two studies analyzed by sex. The results showed no significant correlation between *ADAM12* rs1044122 and OA susceptibility in male and female patients. Subgroup analysis with an average age of 60 years indicated that the rs1044122 polymorphism and susceptibility to OA may not be significantly related to age, as shown in Table 3.

#### FPRP results

We calculated the value of FPRP under a series of prior probability conditions to determine whether there is a real association between *ADAM12* polymorphism and OA. The FPRP results show (Table 4) that when the prior probability is 0.25. The FPRP values of all four genetic models of rs1871054 were less than 0.2. Similarly, with a prior probability of 0.1, the FPRP values of the four genetic models of Asian rs1871054 were all less than 0.2. However, when the prior probability is 0.25, only two genetic models with FPRP values of rs1871054 locus for people younger than 60 years old are less than 0.2. This suggests that ADAM12 rs1871054 polymorphism may have a real association with OA susceptibility, and this association is more real in Asian population, which is worthy of further study. However, in those less than 60 years of age, there is a possibility of false positives between Rs1871054 polymorphism and OA susceptibility.

**Table 4:** FPRP analysis of the noteworthy results for ADAM12 polymorphisms

SNP	Subgroup	Genetic model		OR	95% CI (*P*-value)	*P*	Power	Prior probability				
								0.25	0.1	0.01	0.001	0.0001
*ADAM12*												
Rs1871054	Overall		Dominant model	1.26	1.05–1.51	0.013	0.970	**0.037**	**0.103**	0.557	0.927	0.992
			Recessive model	1.68	1.13–2.51	0.011	0.290	**0.105**	0.260	0.794	0.975	0.997
			Allelic model	1.34	1.05–1.71	0.019	0.818	**0.064**	**0.170**	0.693	0.958	0.995
			CC versus GG	1.62	1.05–2.50	0.031	0.364	**0.195**	0.420	0.889	0.988	0.999
	Asian		Dominant model	1.52	1.20–1.92	0.001	0.456	**0.003**	**0.009**	**0.088**	0.493	0.907
			Recessive model	2.08	1.35–3.20	0.001	0.068	**0.036**	**0.102**	0.555	0.926	0.992
			Allelic model	1.61	1.25–2.08	0.001	0.294	**0.003**	**0.008**	**0.083**	0.477	0.901
			CC versus GG	2.21	1.52–3.22	< 0.001	0.022	**0.005**	**0.015**	**0.142**	0.625	0.943
	Age < 60		Dominant model	1.31	1.02–1.68	0.033	0.857	**0.105**	0.260	0.794	0.975	0.997
			Recessive model	1.91	1.07–3.40	0.029	0.206	0.289	0.549	0.931	0.993	0.999
			Allelic model	1.39	1.01–1.92	0.286	0.678	**0.168**	0.378	0.870	0.985	0.999
			CC versus GG	1.74	1.00–3.02	0.049	0.299	0.330	0.596	0.942	0.994	0.999

Statistical significance values are shown in bold

*CI* Confidence interval; *OR* Odds ratio; FPRP values < 0.2 were considered significant

## Discussion

The genetic factors of OA are mediated by both gene and/or protein expression networks. Among them, coding RNAs and non-coding RNAs have been confirmed to participate in and affect the development of OA [44–46], such as mRNAs, microRNAs, long non-coding RNAs, etc. Several transcription and growth factors (including SOX family members SOX9, L-SOX5, and SOX6) [47], bone morphogenetic proteins, and transforming growth factor β are involved in the modulation of chondrogenesis [48]. There are also epigenetic mechanisms, including DNA methylation and histone modification, that add additional levels of regulation to the evolution of OA [49]. In the mRNA, *ADAM12* is a Zn^2+^-dependent metalloproteinase that may be involved in various cell interactions and biological processes that regulate cell responses [50]. The importance of the *ADAM12* gene in OA has been confirmed by many studies, and its expression is increased to varying degrees [51, 52]. It promotes cell proliferation, differentiation, and migration through outdomain shedding of mesangial epidermal growth factor receptor ligands [53]. Studies have shown that both mRNA and protein levels of *ADAM12* are increased in the synovial tissues of OA-associated synovitis [54]. To explore the gene polymorphisms related to the susceptibility of knee arthritis, we can identify susceptible populations by their genetic phenotype [55] to enable targeted prevention and treatment. However, existing reports are inconclusive. rs3740199 is associated with the risk of OA in a recessive model, but not with rs1871054 [42]. Kerna believed that rs3740199 CC is homozygous for the development of patellofemoral OA [37]. However, Shin et al. found no association between rs3740199 and knee OA. Another study found that rs1871054 and rs1044122 were significantly correlated with knee arthritis, especially in female patients, and those with haplotype CC were more prone to bilateral knee arthritis [24]. Valdes and Kerna reported that the rs1871054 polymorphism was not significantly associated with knee arthritis [35, 37], but four studies reported that rs1871054 was associated with knee arthritis [24–26, 39].

Based on these different conclusions, five studies analyzed the correlation between *ADAM12* polymorphisms and OA. Hu et al. [56] included 10 studies that suggested that rs1871054 is associated with knee arthritis risk. However, there was no significant correlation between rs3740199 and rs1278279, and there were no relevant data in the included studies. Jung et al. [57] included six studies and reported that the risk of knee OA was correlated with rs3740199 and rs1871054. Wu et al. [27] included eight studies and concluded that *ADAM12* rs3740199 polymorphism is related to susceptibility in male patients. Khan et al. [58] included 11 studies, 3 of which had no clinical data, and concluded that the risk of knee arthritis was correlated with rs3740199 and rs1871054, but not with rs1044122 and rs1278279. Lv et al. [21] included seven studies, and their conclusion was similar to that of Hu et al.; namely, that the genetic effect of the rs1871054 polymorphism was stronger in Asian populations than that in European populations. The results of these meta-analyses differ greatly. In recent years, several studies have explored the relationship between these two.

This meta-analysis aimed to explore the relationship between *ADAM12* polymorphisms and the risk of OA. To date, four important polymorphisms of *ADAM12* (rs3740199, rs1871054, rs1278279, and rs1044122) have been associated with OA. The study included 3332 patients with OA in the case group and 5108 patients without OA in the control group. The results show that in dominant, recessive, allelic, and homozygous models, rs1871054 polymorphism was associated with OA. In the subgroup analysis, we found that rs1871054 was associated with OA in Asian populations, but there was no statistical significance in European populations. In addition, people younger than 60 years who carry the C allele may be highly susceptible to the disease, and this conclusion may be a false positive. These differences may be the result of the gene-environment or gene–gene interactions, but the number of studies included is limited. However, the polymorphisms rs3740199, rs1044122, and rs1278279 in *ADAM12* were not significantly correlated with OA, and the results showed no significant correlation after excluding heterogeneity.

In conclusion, *ADAM12* rs1871054 may be a predictor of OA, and individuals carrying the C allele may be highly susceptible to this disease; in addition, the Asian population may also show high susceptibility. Furthermore, rs3740199, rs1044122, and rs1278279 may not be predictors of OA. Although some studies have been published on the rs3740199, rs1871054, rs1044122, and rs1278279 polymorphisms of the *ADAM12* gene and susceptibility to OA, the conclusions are not uniform and lack the support of sufficient homogeneity and large samples in research. If consistent conclusions can be drawn, it will be of great significance for the detection and treatment of OA. Heterogeneity may also be increased due to the different genotyping methods of the included studies. Age and sex differences in region, population, and included population are also important factors affecting the results. Due to the small number of included articles, it is impossible to analyze various interfering factors, and there is certain heterogeneity; therefore, the results need to be carefully interpreted. Whether *ADAM12* polymorphisms are related to susceptibility to OA needs to be supported by higher quality case–control studies with larger samples to provide more effective evidence for the pathogenesis and treatment of OA.

## Data Availability

For further enquiries about the relevant original materials of this article, please consult the corresponding author.
