# Correction: The association between *ADAM12* gene polymorphisms and osteoarthritis: an updated meta-analysis

**DOI:** 10.1186/s13018-023-03685-w

**Published:** 2023-03-25

**Authors:** Su Yang, Yue-peng Wang, Xi-yong Li, Peng-yong Han, Peng-fei Han

**Affiliations:** 1 Department of Orthopedics, Heping Hospital Affiliated to Changzhi Medical College, Changzhi, Shanxi China; 2 Department of Graduate School, Changzhi Medical College, Changzhi, Shanxi China

**Correction: Journal of Orthopaedic Surgery and Research (2023) 18:149** 10.1186/s13018-023-03626-7

Following publication of the original article [1], the author would like to update the affiliations of all the authors as follows.

Department of Orthopedics, Heping Hospital Affiliated to Changzhi Medical College, Changzhi, Shanxi, China.

The original article has been corrected.
